# Comparative Physiology

**Published:** 1891-01

**Authors:** Hugh Hagan


COMPARATIVE PHYSIOLOGY.—A Text-book for students and practitioners of medicine, written by Wesley Mills, M. A. M., D.D. V. S., Professor of Physiology in the Faculty of Medicine, and the Faculty of Comparative Medicine and Veterinary science, of McGill University, Montreal. Author of a Text-Book of Annual Physiology, etc. Published by D. Appleton & Co., New York.

This work, though one of many, on the subject of comparative physiology, is destined to take a position in the foremost ranks of authorities on comparative physiology, for the subject is deftly handed by a man well calculated to elucidate the maze of nature's laboratory, and her wonderful modes.

The work, though not voluminous, is concise, lucid, and well presented. His subdivisions are all scientifically made and presented in such plain, though classic English, and well made plates, that what he lacks in voluminousness, is fully compensated for, by masterly cuts and diagrams, with which his work abounds.

His opening chapter on Biology contains as much information as many others do in volumes. Then as he goes on to deal with reproduction and Embryology, the reader is impressed with the potency of the claim that these two subdivisions of physiology have to more generaland profounder study, as a means to the proper understanding of nature in her fullness and maturity.

Each succeeding chapter is treated of in the same masterly and scholarly manner, and when the reader will have turned the last page, he will say that the knowledge acquired by the study of these 600 pages is well worth the time and energy given.

I take pleasure in recommending the work alike to the profession and the laity. HUGH HAGAN.

